# The AndroCoV Clinical Scoring for COVID-19 Diagnosis: A Prompt, Feasible, Costless, and Highly Sensitive Diagnostic Tool for COVID-19 Based on a 1757-Patient Cohort

**DOI:** 10.7759/cureus.12565

**Published:** 2021-01-07

**Authors:** Flavio A Cadegiani, Ricardo A Zimerman, Bruno Campello de Souza, John McCoy, Rute Alves Pereira e Costa, Carlos Gustavo Wambier, Andy Goren

**Affiliations:** 1 Diabetes and Endocrinology, Corpometria Institute, Brasilia, BRA; 2 Department of Clinical Endocrinology, Federal University of São Paulo, São Paulo, BRA; 3 Applied Biology Inc, Irvine, USA; 4 Internal Medicine, Santa Casa de Porto Alegre, Porto Alegre, BRA; 5 Pan American Health Organization (PAHO), Brasília, BRA; 6 Department of Management Sciences, Federal University of Pernambuco, Recife, BRA; 7 Research: Centro Nacional de Pesquisa em Energia e Materiais, Universidade Estadual de Campinas (Unicamp), Campinas, BRA; 8 Department of Dermatology, The Alpert Medical School of Brown University, Providence, USA

**Keywords:** covid-19, sars-cov-2, pandemic, diagnosis, covid-19 diagnosis, anosmia, ageusia, clinical diagnosis of covid-19, rtpcr, rtpcr-sars-cov-2

## Abstract

Introduction

A major barrier for successful therapeutic approaches for COVID-19 is the inability to diagnose COVID-19 during the viral replication stage, when drugs with potential antiviral activity could demonstrate efficacy and preclude progression to more severe stages. Reasons that hamper an earlier diagnosis of COVID-19 include the unspecific and mild symptoms during the first stage, the delay in the diagnosis and specific management caused by the requirement of a real-time reverse transcriptase-polymerase chain reaction (RT-PCR) for SARS-CoV-2 for the diagnosis of COVID-19, and the insufficient sensitivity of the RT-PCR-SARS-CoV-2, converse to what is recommended for a screening test during an outbreak. More sensitive and earlier diagnostic tools for COVID-19 should be unraveled as a key strategy for a breakthrough change in the disease course and response to specific therapies, particularly those that target the blockage of viral shedding. We aimed to create an accurate, sensitive, easy-to-perform, and intuitive clinical scoring for the diagnosis of COVID-19 without the need for an RT-PCR-SARS-CoV-2 (termed The AndroCoV Clinical Scoring for COVID-19 Diagnosis), resulting from a 1,757 population cohort, to eventually encourage the management of patients with a high pre-clinical likelihood of presenting COVID-19, independent of an RT-PCR-SARS-COV-2 test, to avoid delays and loss of appropriate timing for potential therapies.

Methods

This is a post-hoc analysis of clinical data prospectively collected of the Pre-AndroCoV and AndroCov Trials, which resulted in scorings for the clinical diagnosis of COVID-19 based on the likelihood of presenting with actual COVID-19 according to the number of symptoms, presence of anosmia, and known positive household contact. Sensitivity, specificity, positive predictive value, negative predictive value, positive likelihood ratio, and accuracy were calculated for subjects screened in two different periods and both periods together, for females, males, and both, in a total of nine different scenarios, according to combinations of one, two, or three or more symptoms or the presence of anosmia in subjects without known positive household contacts, and no symptoms, one, two, or three or more symptoms, or presence of anosmia or ageusia in subjects with known positive household contacts. Scorings that yielded the highest pre-test probability, sensitivity, and accuracy were selected.

Results

Of the 1,757 patients screened, 1,284 were diagnosed with COVID-19. The scoring that required: (1) two or more symptoms, or anosmia or ageusia alone, for subjects without known contact; or (2) one or more symptoms, including anosmia or ageusia alone, when with known positive contacts presented the highest accuracy (80.4%) among all combinations attempted, and higher sensitivity (85.7%) than RT-PCR-SARS-CoV-2 commercially available kit tests.

Conclusion

The AndroCoV clinical scoring for COVID-19 diagnosis was demonstrated to be a feasible, easy, costless, and sensitive diagnostic tool for the clinical diagnosis of COVID-19. Because the clinical diagnosis of COVID-19 avoids delays in specific treatments, particularly for high-risk populations, prevents false-negative diagnosis, and reduces diagnostic costs, this diagnostic tool should be considered as an option for COVID-19 diagnosis, at least while SARS-CoV-2 is the prevailing circulating virus and vaccination rate is below the required for herd immunity.

## Introduction

While the COVID-19 pandemic has affected millions of people worldwide, its early stage remains poorly elucidated [1]. Our inability to better characterize the exact pathophysiological mechanisms, as well as its clinical and biochemical presentation, during the first days of the COVID-19 disease course may be explained by the challenging and peculiar characteristics of SARS-CoV-2 and its consequent disease (COVID-19).

First, the exact transmission patterns and incubation period remain not entirely unveiled and may vary according to viral mutations, which precludes precise estimations of the disease timing within each infected subject. Second, symptoms in the first stage of COVID-19 are essentially unspecific since clinical manifestations can resemble those usually present in upper respiratory tract infection (URTI), dengue fever, and/or gastrointestinal (GI) infections [2-5], reducing the chances of a subject being clinically suspected for COVID-19 before more severe stages, unless there was contact with a household known to be positive for COVID-19. Third, researches on COVID-19 have mainly focused on approaches to reduce mortality in already severely affected COVID-19 subjects [1]. Forth, in contrast to the first stage, the pathophysiology of the second stage of COVID-19 has been thoroughly described as being fundamentally mediated by overreactive, dysfunctional inflammatory responses, including the phenomena of cytokine storm [1]. Conversely, virological activity in the second stage becomes trivial and does not play an important role in the typical clinical state observed during this phase. Despite the elucidation of the prevailing pathophysiological mechanisms during the second stage, pharmacological approaches targeting antiviral effects have been persistently tested for this stage, when efficacy for the improvement of COVID-19 outcomes would be unexpected, while studies with potential antiviral agents during the viral replication stage, i.e., during actual early and mild COVID-19, in an outpatient setting (before hospitalization), are lacking [6-8]. The apparent inability to change the COVID-19 course before hospitalization due to the supposed lack of effective pharmacological approaches to early COVID-19 likely demotivated basic and clinical research at this stage of the disease.

In addition to the challenges in the management of COVID-19 during its earliest stages due to the inherent characteristics of SARS-CoV-2 while many subjects are under-suspected due to a lack of the typical clinical characteristics of early COVID-19, whenever COVID-19 is suspected, the prerequisite of a positive real-time polymerase chain reaction (RT-PCR) for SARS-CoV-2 (RT-PCR-SARS-CoV-2) for the diagnosis of COVID-19 delays its diagnosis and specific treatments.

Although RT-PCR-SARS-CoV-2 remains the gold standard diagnostic test for COVID-19, its sensitivity demonstrates wide variations between different commercially available kit tests [9-11] and is overall insufficient for screening purposes. The overwhelming number of false-negative RT-PCR-SARS-CoV-2 tests [12-18] precludes the diagnosis of COVID-19 since rtPCR-SARS-CoV-2 is the only widely accepted diagnostic tool for COVID-19, allowing progression to severe states in several undetected subjects.

The inability to detect COVID-19 in earlier stages is a major concern, particularly for subjects at higher risk for severe COVID-19, including those above 60 years old, with obesity, hypertension, and type 2 diabetes mellitus (T2DM), with preexisting pulmonary conditions, males with androgenetic alopecia (AGA), and females with hyperandrogenism [19-22].

Finally, it seems unfeasible to detect COVID-19 in the earliest days of the disease since subjects are either under-suspected or their diagnoses are delayed due to the necessary positive RT-PCR-SARS-CoV-2 for a definitive diagnosis. The lack of diagnostic options for early COVID-19, i.e., during the viral replication period, hampers the possibility to test the efficacy of potential antiviral approaches to COVID-19, which would eventually be the only actual option to change the disease course.

The increased time to diagnosis and the large number of false-negative RT-PCR-SARS-CoV-2 tests that precluded the correct timing for testing specific antiviral approaches for COVID-1 contributed to the lack of randomized control trials (RCTs) conducted during actual early COVID-19. Some RCTs that claimed to have been conducted in mild, early COVID-19 subjects actually presented criteria or signs of COVID-19 in later stages in the majority of the subjects enrolled in these RCTs [6-8], allowing misleading conclusions regarding the efficacy of certain approaches to early COVID-19.

It seems urgent that more sensitive and earlier methods of COVID-19 detection are developed, which could be the key for a breakthrough change in the COVID-19 disease course and response to specific therapeutic strategies since the majority of new molecules and drug repurposing focused on their potential antiviral activity, which would find the most effective results earliest in the disease.

Indeed, in the AndroCoV trial, we considered as suspect COVID-19 subjects presenting any symptom, regardless of contact with a positive household. The change toward more sensitive COVID-19 detection may justify the very few complications in our RCTs, rather than the therapeutic options. This points to the fact that the timing of the COVID-19 diagnosis and management may play a more important role than which therapeutic options to be employed, as demonstrated in one of our studies [5].

Considering that: 1) clinical or radiological criteria for other viral infections is sufficient for the diagnosis of these infections; 2) the need for a positive RT-PCR-SARS-CoV-2 for the diagnosis of COVID-19 is a barrier in terms of cost and diagnostic delays; 3) infections caused by other agents are unlikely to occur during the pandemic when SARS-CoV-2 is the prevailing virus circulating and other infections are effectively prevented by the spread use of masks; 4) since SARS-CoV-2 is the prevailing virus during the pandemics, a range of different and unspecific symptoms are more likely to be caused by SARS-CoV-2, rather than any other microorganism; and 5) for screening purposes, diagnostic tools more sensitive than RT-PCR-SARS-CoV-2 are highly recommended, our objective was to propose a clinical scoring for the diagnosis of COVID-19 with high sensitivity, accuracy, and higher pretest probability than the RT-PCR-SARS-CoV-2, validated in a large population sample, in order to encourage the management of patients with a high pre-clinical likelihood of presenting COVID-19, at least during the pandemic, without the need of a confirmatory RT-PCR-SARS-COV-2 result.

This article was previously posted to the ResearchGate preprint server on December 24, 2020 [23].

## Materials and methods

Design

This is a post-hoc analysis of clinical data collected prospectively from subjects screened for COVID-19 for the Pre-AndroCoV trial, the AndroCov trial, and without participating in any of the RCTs but following the same clinical protocol [2-5].

The data analyzed included the number of symptoms presented prior to the diagnosis of COVID-19, the presence or absence of anosmia or ageusia, and whether there were positive household contacts before the diagnosis.

The following symptoms were actively searched and considered for the diagnosis of COVID-19: 1) manifestations specific to COVID-19: hyposmia, anosmia, dysgeusia, or ageusia; 2) symptoms typically present in dengue fever (dengue fever-like syndrome): myalgia, arthralgia, upper back pain, conjunctival hyperemia, pre-orbital pain; 3) symptoms of upper respiratory tract infection (URTI) (URTI-like syndrome): nasal congestion, rhinorrhea, dry cough, self-reported perception of “sinusitis,” or self-reported perception of “sore throat”; 4) symptoms of acute gastroenteritis (GE) (GE-like syndrome): nausea, vomiting, or abdominal pain; 5) unspecific symptoms: lower back pain, leg pain, feverish, fatigue, weakness, dizziness, and headache. For subjects that experimented with symptoms frequently or presented prior to any suspicion of COVID-19, changes in the patterns of these manifestations were required to be counted as a symptom.

A subset of 200 subjects was presumedly diagnosed for COVID-19 based on the resulting clinical scoring that demonstrated the highest accuracy and a pre-test probability higher than RT-PCR-SARS-CoV-2 sensitivity and was followed prospectively and treated accordingly. All 200 patients underwent a first RT-PCR-SARS-CoV-2, and those with negative results underwent a second RT-PCR-SARS-CoV-2 between 24 and 72 hours later than the first one. 

Scenarios for the clinical diagnosis of COVID-19 were tested for precision-related statistical parameters. Scenarios tested for the preciseness of the COVID-19 diagnosis included combinations of the following parameters: when one, two, or three or more symptoms were present, or when anosmia or ageusia was presented, in the presence or absence of known positive households. Scenarios were tested for three periods, including two distinct periods, and a third period that encompassed the two periods evaluated distinctly from each other. The first period comprised the observational study of the AndroCoV Trial (pre-AndroCoV Trial), between May 2020 and July 2020, and the second period comprised the AndroCoV RCTs and the follow-up of untreated patients between July 2020 and December 2020. Each scenario was tested for the three periods, for males, females, and overall subjects.

Accuracy and sensitivity were compared for different scenarios, and those that disclosed the highest values were employed as the basis for the development of the clinical scorings in a manner to allow the clinical diagnosis of COVID-19 when a minimum number of points was reached in a manner that reflected the scenarios with the highest accuracy and sufficient sensitivity.

Statistical analysis

Sensitivity, specificity, pretest probability, positive and negative predictive value, accuracy, and positive likelihood ratio were calculated. For the calculations, screening of subjects without symptoms and without known positive household contacts was not considered since there is no justification to screen for COVID-19 in this population.

## Results

Subjects 

In total, 1,757 subjects were screened for COVID-19, including 1,557 analyzed retrospectively and 200 that were presumedly diagnosed for COVID-19 and followed up prospectively. Of the 1,757 subjects, 1041 were males (59.2%) and 716 were females (40.8%). In the first period, 755 patients were screened (42.7%), including 413 males (54.7% of 755 patients) and 342 females (45.3% of 755 patients). In the second period, 1,002 patients were screened (57.3%), including 628 males (62.7% of 1002 patients) and 374 females (37.3% of 1002 patients). No non-binary or non-cissexual subjects were screened.

Of the 1,757 subjects screened, 1,284 (73.1%) were diagnosed with COVID-19, including 585 subjects enrolled in the observational study (pre-AndroCoV trial), 94 in the spironolactone arm of the AndroCoV trial (SPIRO AndroCoV-Trial), 138 in the dutasteride arm of the AndroCoV trial (DUTA AndroCoV-Trial), 169 patients in the Proxalutamide arm of the AndroCoV Trial (PROXA AndroCoV-Trial), and 198 subjects followed as untreated controls.

Analysis of the scenarios

**Figure 1:**
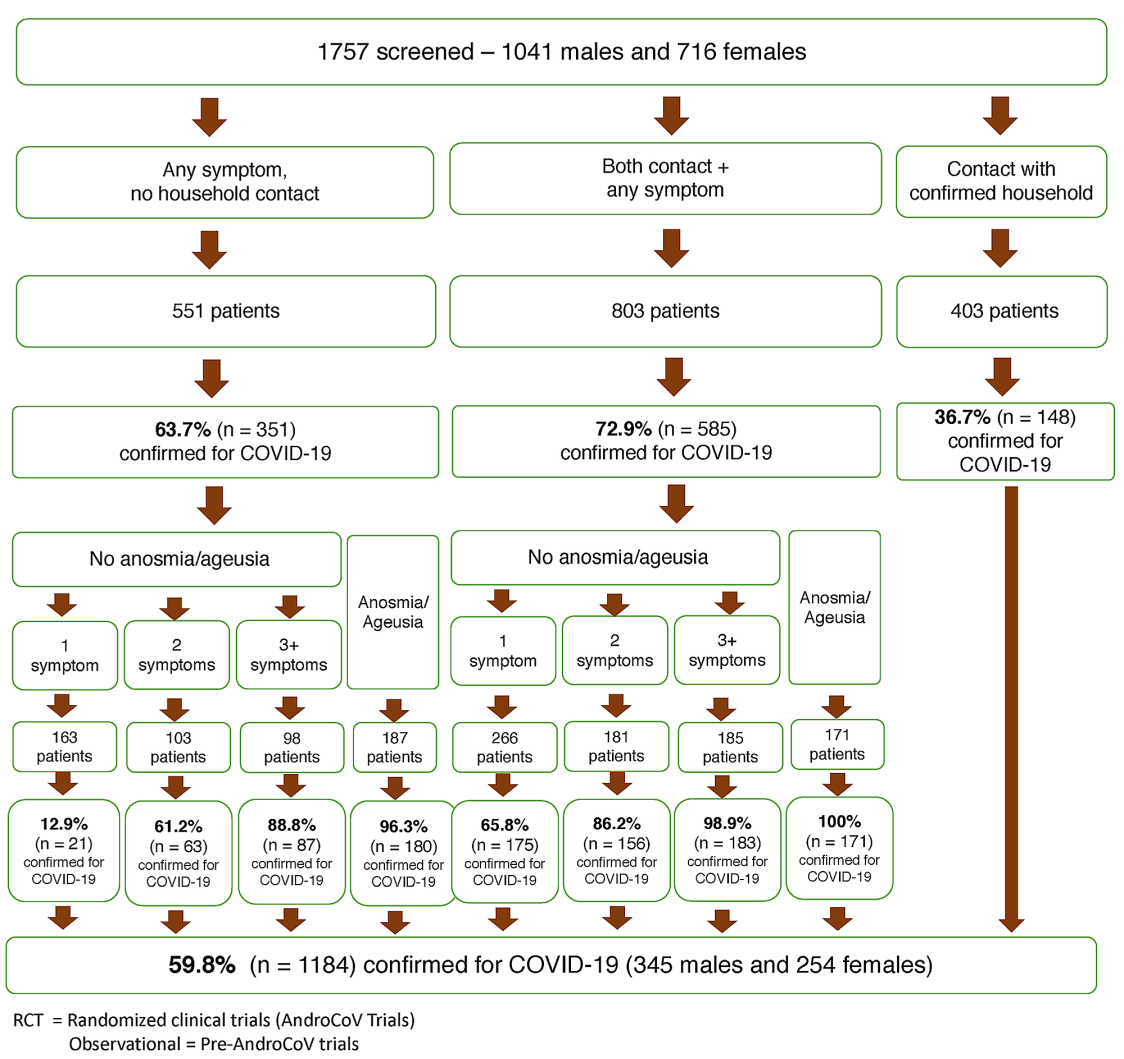
Positivity rates for RT-PCR-SARS-CoV-2 according to clinical characteristics and contact with a positive household RT-PCR-SARS-CoV-2 = real-time polymerase chain reaction for SARS-CoV-2

The positivity rates of the RT-PCR-SARS-CoV-2 tests according to the number of symptoms, presence of anosmia or ageusia, and contact with a positive household are displayed in Figure 1 (the full descriptions of scenarios with males, females, or both, in the first period, second period, or both periods together are shown in Appendix 1). We found positivity rates above 60% when at least two symptoms, not including anosmia or ageusia, were present irrespective of household contact, above 80% when at least one symptom was present in subjects with a known positive household contact, or three or more symptoms were present without a known positive contact, and above 95% when anosmia or ageusia was present, irrespective of previous known contact with positive households, or when three or more symptoms were present with a known positive household. All patients with anosmia or ageusia and known positive household were positive for COVID-19.

**Figure 2:**
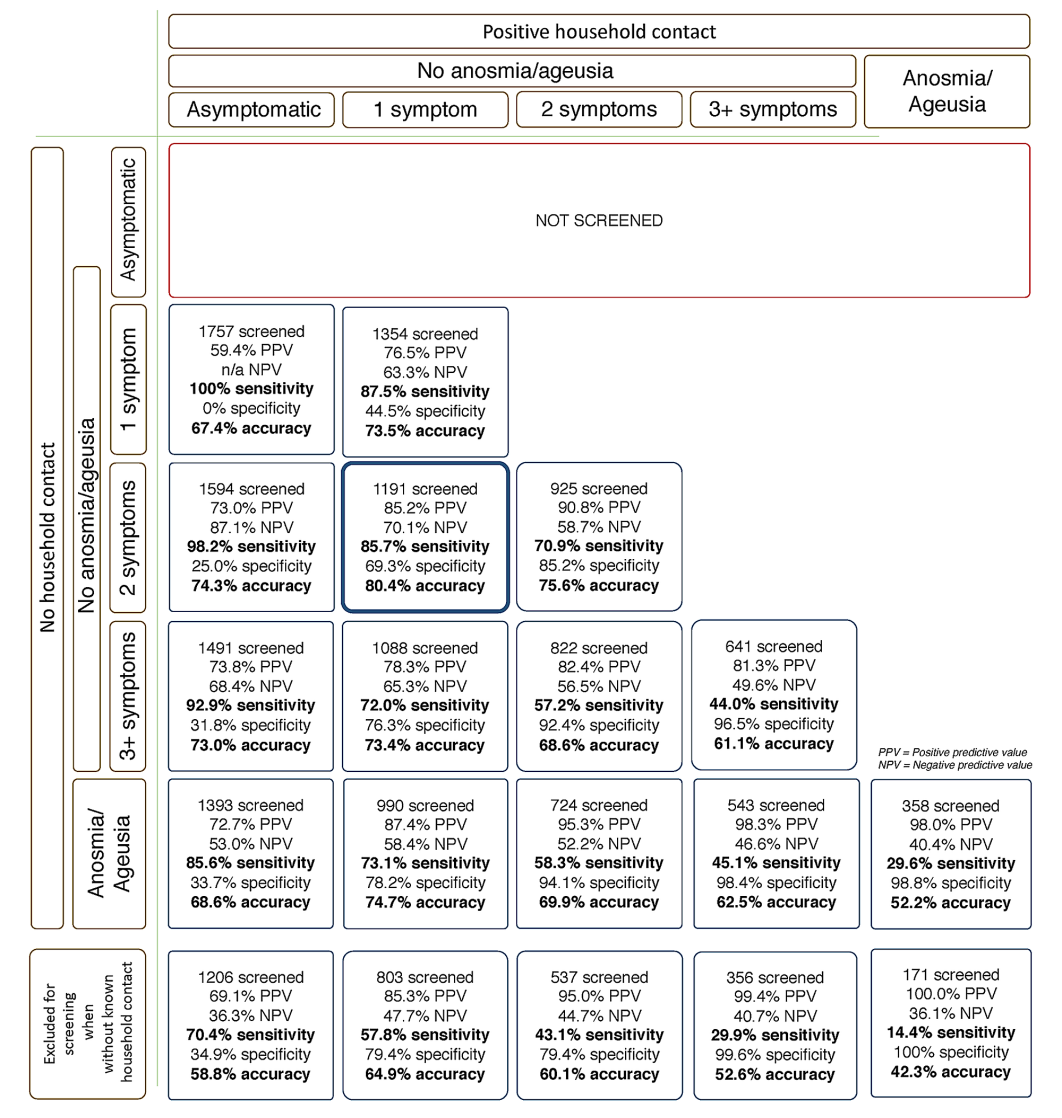
AndroCoV clinical diagnostic scoring combinations

Figure 2 presents the sensitivity, specificity, accuracy, positive predictive value (PPV), and negative predictive value (NPV) values to detect COVID-19 using clinical scorings in different combinations, according to the number of symptoms, presence of anosmia or ageusia, and known positive households. (The full characterization of each scenario is disclosed in Appendix 2, and the full tables with the number of subjects encompassed in each combination, as well as the number of true positives, true negatives, false positives, false negatives, sensitivity, specificity, PPV, NPV, accuracy, and positive likelihood ratio values for each of the nine possible scenarios are shown in Appendix 3.)

Combinations that demonstrated sensitivity and accuracy above 80% and 70%, respectively, i.e., a pretest probability higher than the RT-PCR-SARS-CoV-2 sensitivity, were those that required: 1) one or more symptoms with or without known positive household; 2) two or more symptoms or anosmia or ageusia without known positive household or contact with a known positive household with or without symptoms; 3) two or more symptoms or anosmia or ageusia without known positive household or one or more symptom with known positive household; 4) three or more symptoms or anosmia or ageusia without known positive household or contact with a known positive household with or without symptoms; 5) three or more symptoms without known positive household or contact with a known positive household with or without symptoms; or 6) whenever anosmia or ageusia was present, irrespective of known positive household or whenever there was contact with a positive household. Among these combinations, the combination of two or more symptoms without known contact or one or more symptoms with known positive contact presented the highest accuracy (80.4%).

**Figure 3:**
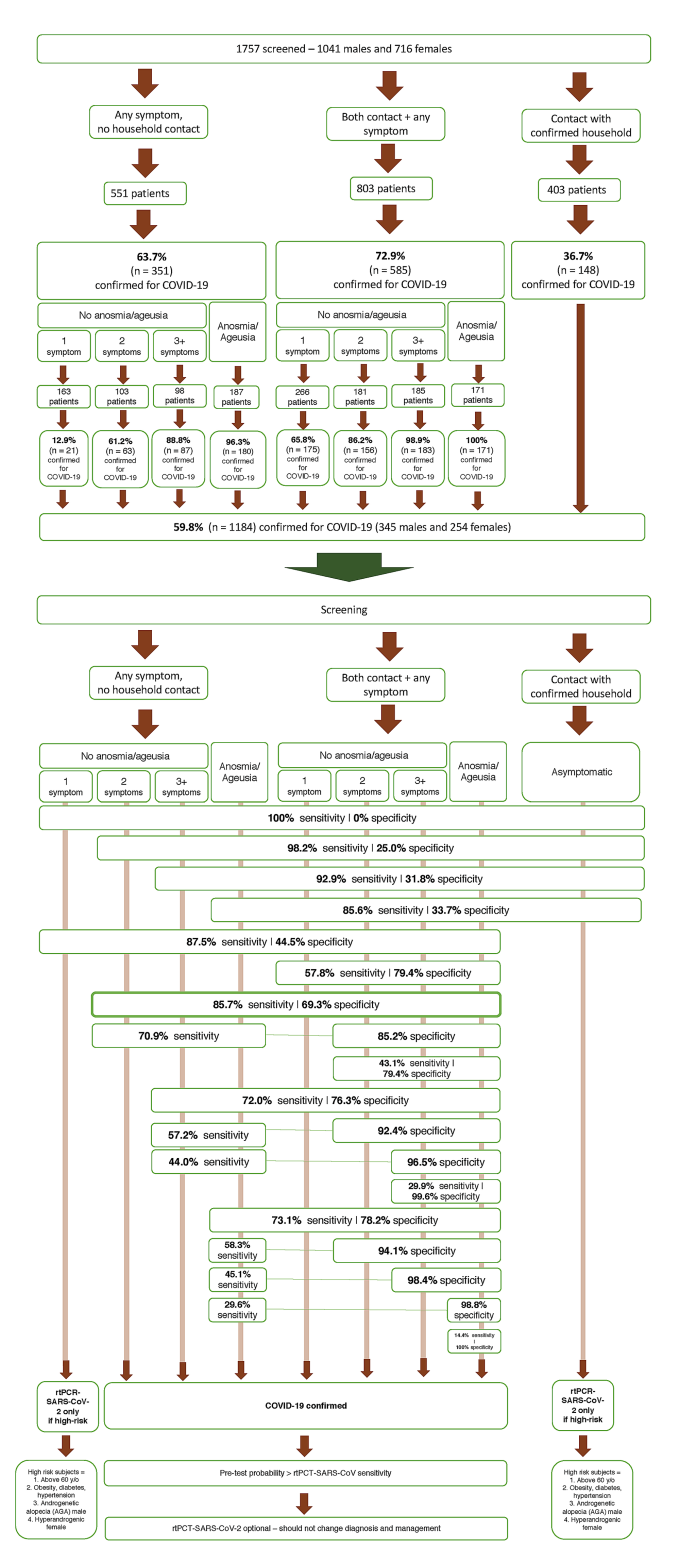
Diagnostic management of COVID-19 according to clinical characteristics and known household contact

Figure 3 displays the recommendations for diagnostic management in cases suspected for COVID-19 according to the number of symptoms, presence of anosmia or ageusia, and contact with positive household based on sensitivity, pre-test probability, and risk of COVID-19 complications, when delays in specific approaches should be avoided. During the COVID-19 pandemic, when two or more symptoms among the listed ones or whenever anosmia or ageusia is present, irrespective of known positive contact, or when one symptom is present after contact with a positive household, COVID-19 can be diagnosed clinically and managed accordingly. Recommendations are valid while SARS-CoV-2 remains the prevailing circulating virus, use of masks remains forced and spread, and vaccination rate is below the expected for herd immunity.

Prospective follow-up

From the diagnostic management proposed in Figure 3, 200 subjects, including 169 from the PROXA Andro-CoV trial and 29 not enrolled in any RCT, were screened using the AndroCoV-derived diagnostic management flowchart and then followed prospectively.

**Figure 4:**
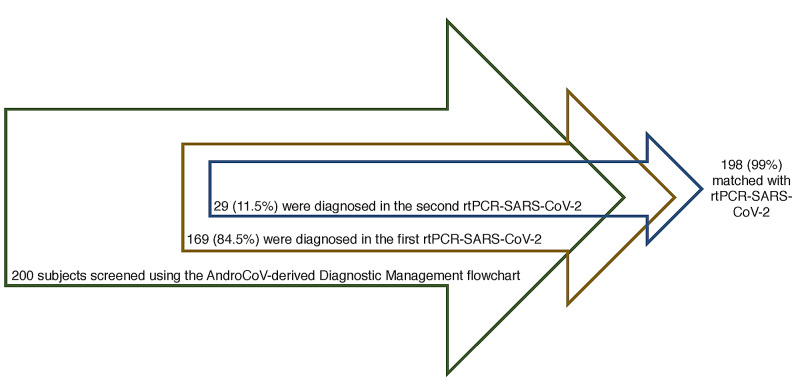
Simplified application of the presumed diagnosis of COVID-19

Of these, 169 (84.5%) were virologically diagnosed in the first RT-PCR-SARS-CoV-2, 29 (11.5%) were diagnosed in the second RT-PCR-SARS-CoV-2, and two (1%) remained negative. Using two consecutive RT-PCRs, the accuracy of the proposed clinical scoring combinations was 99% (Figure 4).

Clinical scoring for COVID-19 diagnosis

**Figure 5:**
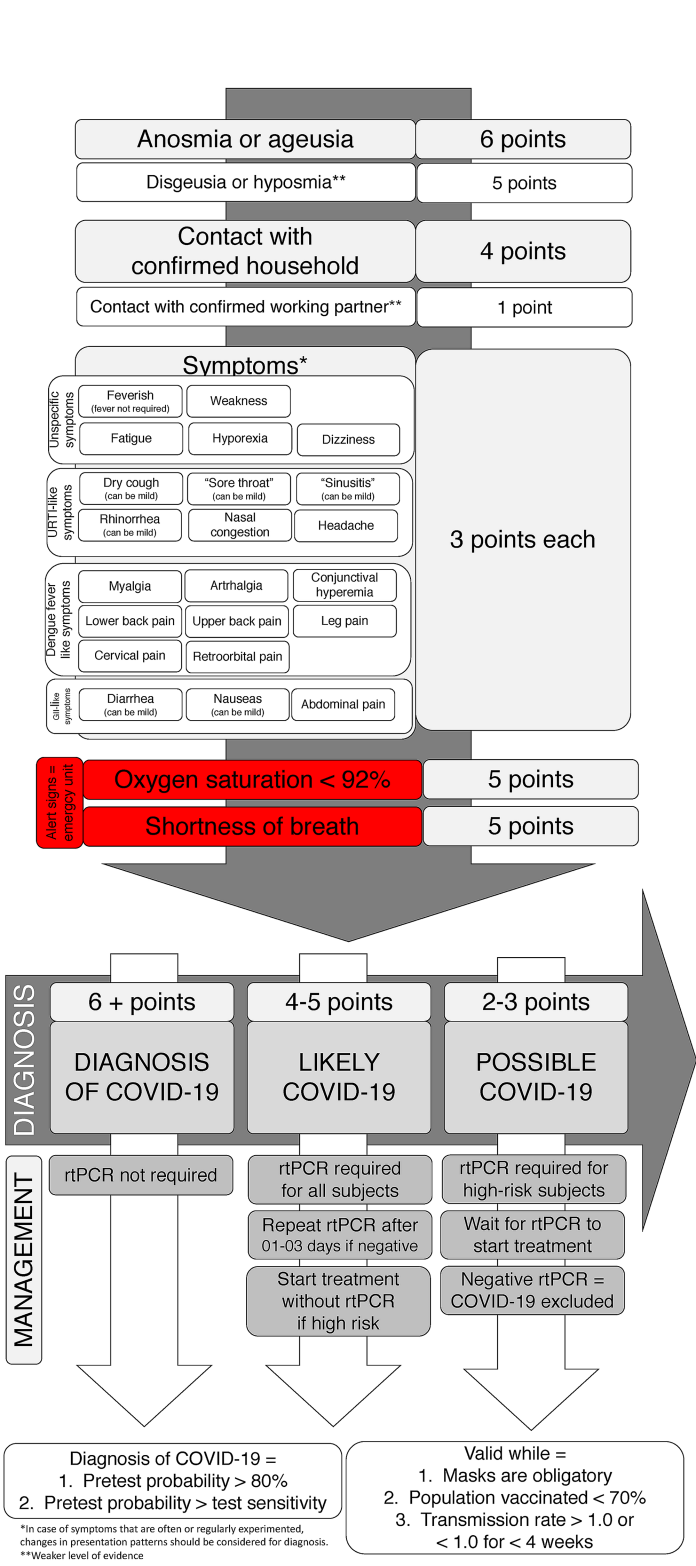
AndroCoV clinical scoring for COVID-19 diagnosis RT-PCR-SARS-CoV-2 = real-time polymerase chain reaction for SARS-CoV-2

From the results presented in Figures 1-5, scoring for the clinical diagnosis of COVID-19 was developed and validated, based on the likelihood of a subject to present COVID-19 according to the number of symptoms, presence of anosmia, and contact with known positive household (Figure 5). 

**Figure 6:**
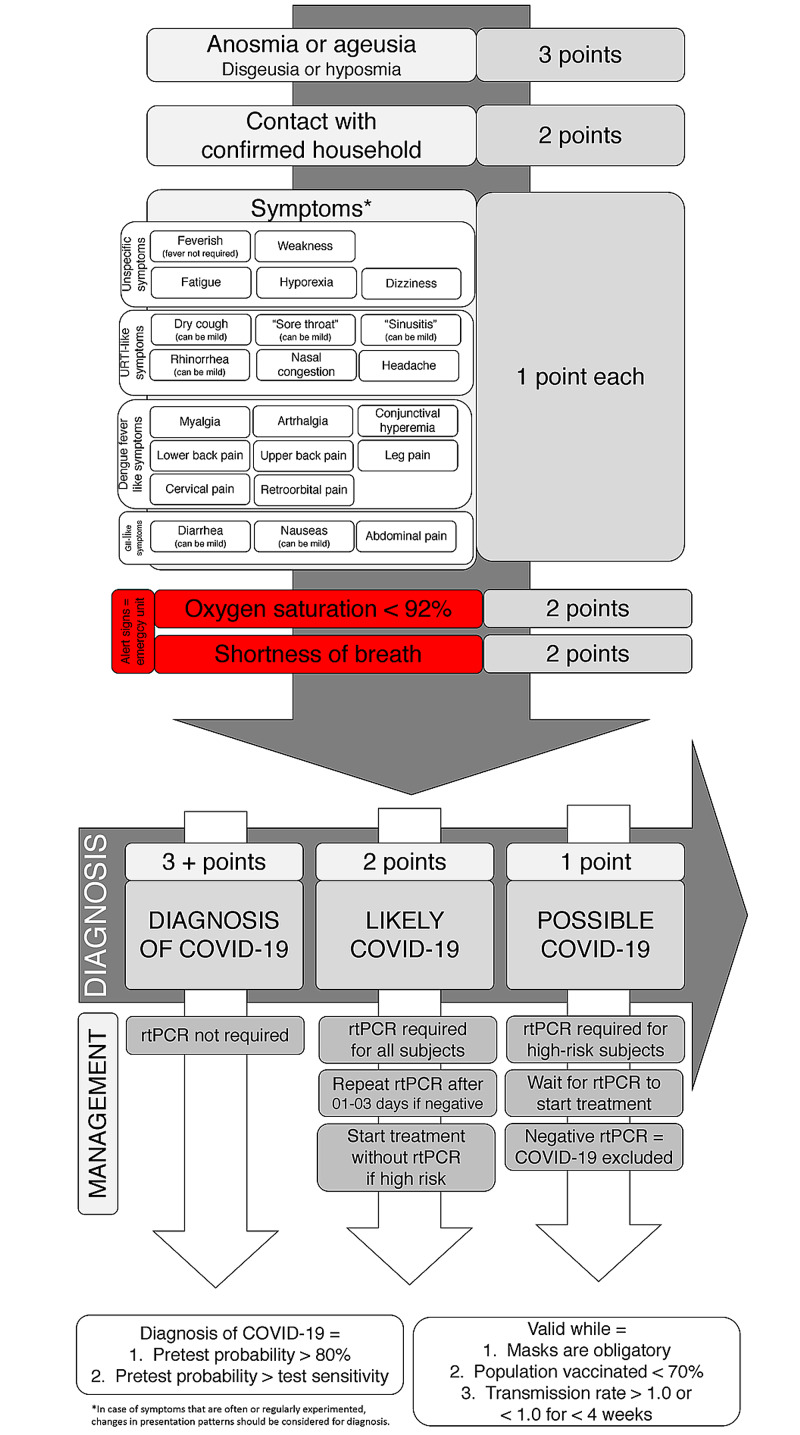
Simplified AndroCoV Clinical COVID-19 diagnosis to be employed for screening in the general population

The term ‘The AndroCoV Clinical Scoring for COVID-19 Diagnosis’ was coined for this scoring. In addition, a simplified scoring with slightly lower accuracy was also developed (‘The Simplified AndroCoV Clinical Scoring for COVID-19 Diagnosis’) (Figure 6).

Characteristics more specifically and critically related to COVID-19 counted as more points. The pointing system that best matched the most accurate combination for clinical diagnosis is displayed in Figure 5 (above 80%). However, an alternative, more simple, easy-to-use, diagnostic flowchart, with acceptable sensitivity (above 70%) and accuracy (above 75%) was also presented and shown in Figure 6. 

For the clinical diagnosis of COVID-19, six or more points are necessary, above which rtPCR-SARS-CoV-2 becomes unnecessary since the pre-test probability is higher than the RT-PCR-SARS-CoV-2 sensitivity. 

When subjects score between four and five points, the diagnosis of COVID-19 is likely, and an RT-PCR-SARS-CoV-2 is required. If the test is negative, a second RT-PCR-SARS-CoV-2 should be performed between one and three days after the first test since the sensitivity of RT-PCR-SARS-CoV-2 tends to be lower at the beginning of the disease. Exceptionally, for high-risk patients, specific approaches or treatments for COVID-19 should be started independently of the RT-PCR-SARS-CoV-2 result and should only be interrupted in case two consecutive tests are negative. Scores of three points or below represent a scenario of possible but not likely COVID-19, and RT-PCR-SARS-CoV-2 is only recommended for high-risk subjects.

As per the scoring and pre-test probability and accuracy, the presence of two or more symptoms, or anosmia or ageusia, or any symptom when with positive household contact are the three key diagnostic possibilities for the clinical diagnosis of COVID-19.

The present scoring is valid while the use of masks is obligatory, the population vaccinated in the region of the subjects screened is below 70%, and transmission rate is > 1.0 or < 1.0 for less than four weeks.

High-risk patients 

The determination of which subjects were at high risk for COVID-19 was based on the medical literature and include subjects above 60 years old, males with androgenetic alopecia (AGA), females with hyperandrogenic states, and those presenting metabolic-related conditions, including obesity, diabetes mellitus, and hypertension (16).

Follow-up

Subjects that do not fulfill criteria for COVID-19 should be reassessed for clinical symptoms and diagnosis of household contacts on a daily basis for three days following the first day of clinical screening, particularly those with four or five points since new symptoms may appear in subjects with actual COVID-19, raising the scoring in the following days and allowing the clinical diagnosis of COVID-19. Whenever an RT-PCR-SARS-CoV-2 is indicated, subjects should be clinically reassessed irrespective of the results, since false-negative RT-PCR-SARS-CoV-2 is correlated with early COVID-19 and milder symptoms.

## Discussion

A clinical diagnosis of COVID-19 based on a sensitive case-detection finds multiple advantages over the desideratum of an RT-PCR-SARS-CoV-2 test, including avoidance of the non-negligible cost of an RT-PCR-SARS-CoV-2 test, avoidance of further delays in potential therapeutical approaches for COVID-19, when the timing may be critical for success, and few severe harms as compared to multiple potential benefits of earlier diagnosis, when ‘overdiagnosis’ is preferred over ‘underdiagnosis’ in the time of the COVID-19 pandemic.

With the present thorough analysis of 1,757 subjects suspected for COVID-19, we found sufficient substantiation to recommend against a mandatory RT-PCR-SARS-CoV-2 for the diagnosis of COVID-19 when COVID-19 is likely. This should reduce screening expenses and, therefore, the inequity caused by the lack of wide access to rtPCR-SARS-CoV-2 kit tests. For patients clinically diagnosed for COVID-19 through our clinical scoring system, the performance of an RT-PCR-SARS-CoV-2 test should be avoided because the clinical diagnosis has demonstrated higher accuracy than commercially available RT-PCR-SARS-CoV-2 kit tests. Indeed, when the diagnosis of COVID-19 is clinically confirmed using the scoring, an RT-PCR-SARS-CoV-2 test may lead to a misdiagnosis, rather than clarification, if performed, in case of a non-insignificant risk of a false-negative result. If the RT-PCR-SARS-CoV-2 result is negative, which may occur in between 10% and 40% of the causes, because of its overwhelming risk of a false-negative result, clinical diagnosis, rather than the test result, must be considered.

To overcome false-negative RT-PCR-SARS-CoV-2 that may lead to the loss of timely detection of subjects developing severe COVID-19, we proposed for moderately suspected patients, a second consecutive RT-PCR-SARS-CoV-2 to be repeated between 24 and 72 hours after the first test since more than 80% of those with a first negative RT-PCR-SARS-CoV-2 showed a positive result when performed again.

A clinical and early diagnosis of COVID-19 is remarkably critical for subjects at higher risk to develop severe COVID-19. In particular, elders may present lower sensitivity for an RT-PCR-SARS-CoV-2 test while their clinical presentation may not always be as typical as the already unspecific symptoms usually presented in COVID-19. This population could be especially benefited from the clinical diagnosis of COVID-19 to prevent further progression to more severe states.

Additional interesting findings were unveiled by the present analysis. Approximately one in every seven subjects with COVID-19 (14.4%) had anosmia or ageusia with known household contact, which means that for every seven patients with COVID-19, six will not present anosmia and known contact with positive household. This finding finds importance to drive improvements in policies for the detection of COVID-19. 

The unique characteristics of the virus, including transmission patterns, overall behavior, clinical course, and heterogeneous presentation not only contributed to the occurrence of the COVID-19 pandemic but also to the lack of success of multiple approaches. Accordingly, the peculiarities of SARS-CoV-2 do not allow the determination of an undisputed method of clinical criteria for a COVID-19 diagnosis. However, a clinical diagnostic tool based on suspecting subjects with any sign of potential COVID-19, including symptoms, whether being specific to COVID-19 or not, contact with confirmed household, or both, may be able to detect virtually 100% of the cases. The only non-encompassed group that could miss sensitivity for COVID-19 diagnosis were asymptomatic patients without known confirmed contacts. However, this population is least likely affected and should not be tested randomly.

Shortness of breath and oxygen saturation have a particular correlation in COVID-19. The presence of shortness of breath with oxygen saturation above 92% is more likely due to anxiety induced by COVID-19 than the disease per se. Conversely, ‘happy hypoxia’ is a common phenomenon observed in COVID-19 in which shortness of breath only occurs when oxygen saturation is overtly low (commonly below 80%). However, since the present clinical diagnostic tool aims to counteract the prevailing inertia and underdiagnosis that has likely led to an excessive number of deaths due to COVID-19, we recommend the investigation of shortness of breath, regardless of oxygen saturation levels.

Similarly, although the number of symptoms alone can lead to a large number of false-positive COVID-19 diagnoses, a counterbalance for the highly specific but insufficiently sensitive RT-PCR-SARS-CoV-2 test in the current context of the pandemic is critically needed when high sensitivity must be pursued.

Clinical scoring for the diagnosis of COVID-19: understanding each aspect of the scoring

The prevalence of anosmia or ageusia was lower in the present analysis than in our own data of the RCTs because part of the subjects positive for COVID-19 developed these symptoms after the diagnosis while these symptoms tend to appear later in the early stage of the disease. However, the presence of anosmia or ageusia before the diagnosis is highly specific to COVID-19, alone showing specificity of 98.8% for the COVID-19 diagnosis, irrespective of contact with positive households, and showing to be the most accurate sign for a COVID-19 diagnosis, rather than RT-PCR-SARS-CoV-2. For this reason, one between anosmia or ageusia alone scores six points in the clinical scoring proposed in the present analysis, sufficient for the clinical diagnosis of COVID-19. Hyposmia and dysgeusia are highly specific as well but may suffer interferences of other URTIs, and provide, therefore, five points. Only the rare cases of subjects with anosmia or hyposmia prior to COVID-19 should be excluded from this evaluation.

Contact with a household confirmed for COVID-19 raises the risk of COVID-19 to 50% to 60% if the positive contact was a female and the suspected subject is a male and 20% to 30% if the positive contact was a male and the suspected case is a female, as demonstrated in our analysis of the same set of subjects (2,13-15). Although the risk of transmission is approximately two times higher when transmission occurs from female to male than in the opposite direction, contact with positive households should not count as different points according to the sex of the contact. Females tend to be less severely affected by COVID-19 and present fewer symptoms than males. Hence, the relative importance of a positive contact is higher for suspected females than males, which compensates for the lower risk of being infected by a positive male contact.

Contact with a positive working partner also raises the risk of COVID-19, although less substantially than when living with positive contact. In addition, data on labor transmission are less explored and undisputed. For this reason, a positive working partner counts as one point only.

Except for anosmia and ageusia, since symptoms of COVID-19 are unspecific at the beginning of the disease, each symptom, not restricted to those classically related to COVID-19, counts as three points each. For matching similar sensitivity, two symptoms are sufficient for the clinical diagnosis since the likelihood of being infected by other viruses or bacteria is low while SARS-CoV-2 is the prevailing circulating virus and the use of masks is spread since the transmission of other viruses and bacteria seems to be more effectively prevented by the use of masks than SARS-CoV-2 [18].

Although two unspecific symptoms allow the clinical diagnosis of COVID-19, manifestations resulted from the anxiety generated by the risk of presenting COVID-19 and the inability to differentiate between symptoms commonly experimented by subjects and new-onset symptoms may lead to an overdiagnosis of COVID-19 and should be considered for the scoring when anxiety states related to COVID-19 are detected.

As shown in the scoring system, four points is sufficient to start specific approaches and treatments for high-risk patients. This is particularly important for elders that present an exponential risk with aging and, at the same time, clinical manifestations tend to be less typical and less pronounced until progression to more severe states occurs.

Reduction in the SARS-CoV-2 circulation and high vaccination rate may decrease the accuracy of the proposed clinical diagnosis. When the criteria of spread use of masks and vaccination rate are no longer met, a possible increase from six to eight points for the clinical diagnosis of COVID-19, for example, should be considered, and the present scoring system should be evaluated continuously.

We attempted to compare our clinical scoring for COVID-19 with currently published scorings. However, previous scorings employed biochemical and radiological parameters that required symptoms that occur later in the disease or considered positive epidemiology as relevant as a positive contact [24-25]. To the best of our knowledge, this is the first purely clinical scoring with extensive validation of COVID-19.

Implications of the clinical diagnosis of COVID-19

The earlier diagnosis of COVID-19 enabled by the present clinical scoring should have multiple consequences for the disease course and management. First, policies for the contention of transmission should become more effective. Second, earlier interventions in high-risk populations, which correspond in the majority of the cases to those subjects less likely suspected for COVID-19 because of the lack of overt and usual symptoms, may allow effective and dramatic changes in the disease course and outcomes. Third, the clinical diagnosis of COVID-19 allows the diagnosis while still in the viral replication period, allowing the demonstration of the efficacy of potential antiviral agents, unveiling the still underreported window of opportunity for antiviral approaches, similar to what has been observed for oseltamivir for influenza A, which shows efficacy only if used in the first three days of the disease [26].

Recommendations based on the findings of the present analysis

Whenever a clinical diagnosis of COVID-19 has a higher pre-test probability than the RT-PCR-SARS-CoV-2 test, we recommend the presumed clinical diagnosis of COVID-19 as being sufficient. We recommend against the use of RT-PCR-SARS-CoV-2 if our proposed clinical scoring indicates the diagnosis of COVID-19.

Six points in the AndroCoV clinical scoring for COVID-19 diagnosis has sufficient substantiation to allow the diagnosis of COVID-19 without an RT-PCR-SARS-CoV-2 test. Alternatively, three points in the simplified AndroCoV Clinical Scoring for COVID-19 diagnosis also allows its diagnosis.

The present recommendation is a translation of the fact that subjects with no household positive contact but presenting at least two symptoms among those listed in the flowcharts (Figure 6), anosmia alone, or ageusia alone should be considered as clinically diagnosed with COVID-19. In the case of positive household contact, one symptom is sufficient to fulfill the criteria for the clinical diagnosis of COVID-19. 

Four to five points in the clinical scoring and two points in the simplified model of clinical scoring show the diagnosis of COVID-19 to be likely, and a confirmatory RT-PCR-SARS-CoV-2 is required. Nonetheless, high-risk patients should not depend on the test result to start specific therapeutic approaches. Whenever RT-PCR-SARS-CoV-2 is negative, subjects should be clinically reassessed daily, and a second RT-PCR-SARS-CoV-2 should be performed between one and three days after the first test. For high-risk patients, specific management should only be interrupted in case of two consecutive negative RT-PCR-SARS-CoV-2 tests.

While SARS-CoV-2 remains as the prevailing circulating virus, the use of masks that effectively block bacterial and other viruses infections, and the RT-PCR-SARS-CoV-2 test still misses between 10% and 40% of the diagnosis of COVID-19, allowing further progress to severe states in undiagnosed subjects, the use of an easy and intuitive scoring flowchart for clinical diagnosis of COVID-19 should be considered as a first-line diagnostic tool for COVID-19.

Anosmia or ageusia are symptoms highly specific to COVID-19 and should be considered in the current context as pathognomonic of COVID-19 when acquired during the pandemic.

Since sensitivity to detect COVID-19 using RT-PCT-SARS-CoV-2 varies according to the time of disease and viral load, clinical diagnosis should be preferred over virological methods.

The employment of rtPCT-SARS-CoV-2 as the sole diagnostic method for patients with a pretest probability of COVID-19 above 80% should be considered as misuse of the test, at least while tests do not become more sensitive than the current commercially available test kits.

The determination of the exact pretest probability, above which an RT-PCT-SARS-CoV-2 test becomes unnecessary, is challenging due to the lack of data regarding viral transmission patterns and the actual number of infected subjects. Hence, thresholds should not only depend on the test sensitivity and pretest and posttest probability but also on the consequences of missing a timely diagnosis of COVID-19 with consequent delays in approaches and the lower efficacy of any therapeutic strategy. On the contrary, earlier diagnosis, even if leads to an excessive number of ‘overdiagnoses,’ may find more benefits than harms in the present context.

The AndroCoV clinical scoring for the diagnosis of COVID-19 may be adapted for regional specificities in terms of clinical presentation, transmission rates, and potential viral mutations that occur in different regions.

The present clinical scoring for the diagnosis of COVID-19 should be valid while transmission rate is above 1.0, below 1.0 for less than four weeks, while masks are widely used and when less than 70% of the population is vaccinated for SARS-CoV-2.

COVID-19 was diagnosed only after the end of the viral replication stage due to two consecutive delays of the lack of clinical suspicion at the beginning of the disease, followed by waiting time until the result of the RT-PCR-SARS-CoV-2 test, which precluded potential antiviral agents to demonstrate efficacy. In contrast to COVID-19 diagnosed through RT-PCT-SARS-CoV-2 or chest computed tomography (CT) scan, the clinical diagnosis of COVID-19 based on a more sensitive case-detection allows an earlier and timely diagnosis still during the viral replication stage. Consequently, we strongly encourage the reassessment of the efficacy of drugs with potential antiviral activity when COVID-19 is diagnosed clinically since the diagnosis during viral replication may represent the still underreported window of opportunity for antiviral approaches, in a similar manner of oseltamivir for influenza A.

Limitations

While we based the positivity rates of COVID-19 according to clinical presentation and contact with a positive household on matches with two consecutive results of commercially available RT-PCR-SARS-CoV-2 kit tests, the determination of pre-test probability is imprecise due to the challenging and still largely unclear understanding of COVID-19 transmission patterns. The present scoring system was based on the SARS-CoV-2 transmission dynamics and clinical characteristics of a specific region (Brasilia, Brazil) and may not precisely reflect patterns observed in other regions.

## Conclusions

A clinical diagnosis of COVID-19 based on the presence of two or more symptoms, or anosmia alone, or ageusia alone, or one symptom when in contact with a positive household, which was translated to the AndroCoV clinical scoring for the diagnosis of COVID-19, was demonstrated to be a feasible, fast, costless, and sensitive diagnostic tool for the diagnosis of COVID-19. The clinical diagnosis of COVID-19 avoids delays in specific approaches and precludes missed diagnoses due to the relatively low sensitivity of RT-PCR-SARS-CoV-2 tests and should be considered to become an option for COVID-19 diagnosis for public health policies.

## Figures and Tables

**Figure 7:**
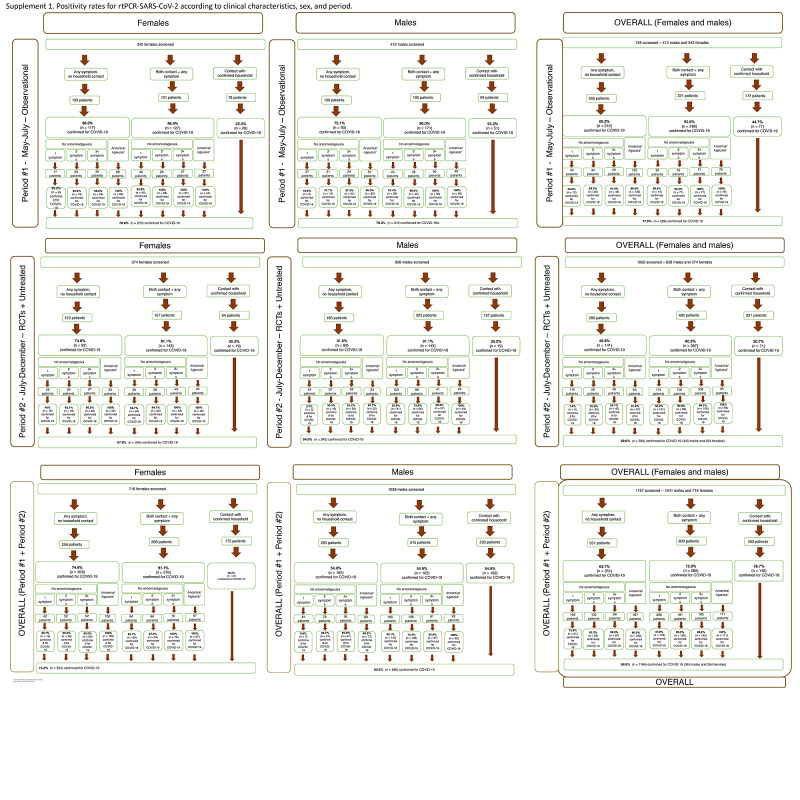
Positivity rates for RT-PCR-SARS-CoV-2 according to clinical characteristics, sex, and period RT-PCR-SARS-CoV-2 = real-time polymerase chain reaction for SARS-CoV-2

**Figure 8:**
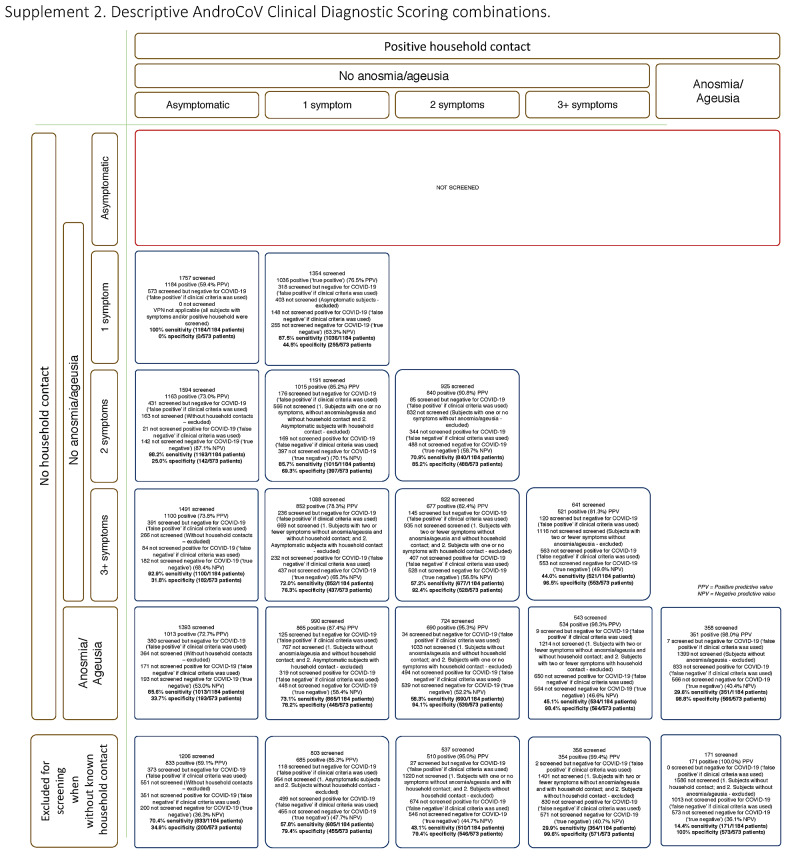
Descriptive AndroCoV clinical diagnostic scoring combinations

**Figure 9:**
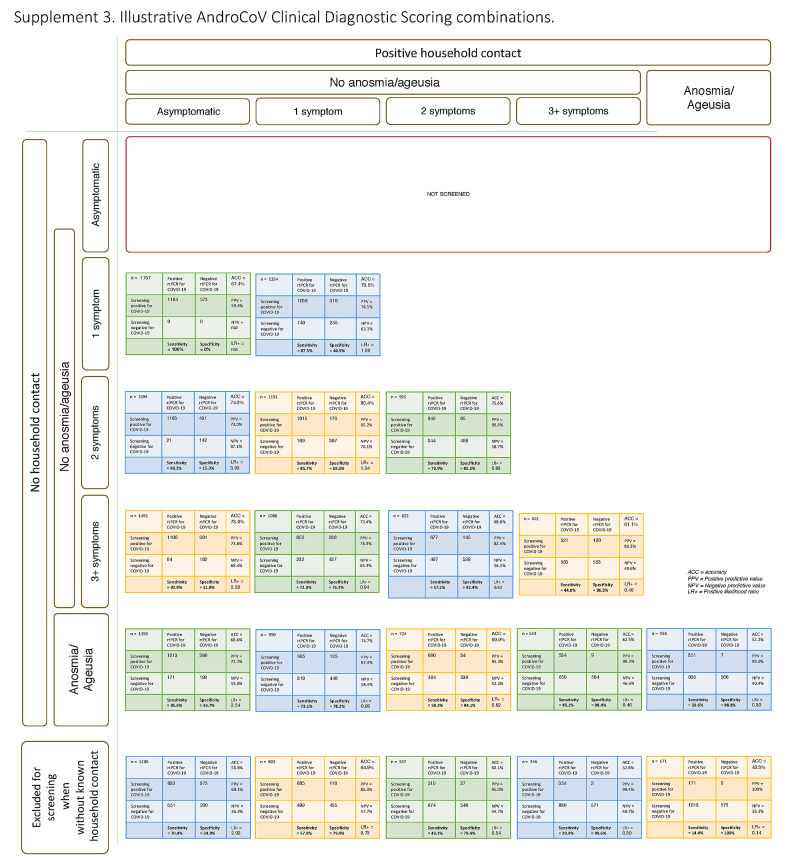
Illustrative AndroCoV clinical diagnostic scoring combinations

## Appendices

Appendix 1 

Appendix 2 

Appendix 3 
